# Long-Term Persistence of Anti-SARS-CoV-2 Antibodies in a Pediatric Population

**DOI:** 10.3390/pathogens10060700

**Published:** 2021-06-04

**Authors:** Ana Méndez-Echevarría, Talía Sainz, Iker Falces-Romero, Beatriz de Felipe, Lucia Escolano, Sonia Alcolea, Lidia Pertiñez, Olaf Neth, Cristina Calvo

**Affiliations:** 1 Pediatrics and Infectious Disease Unit, La Paz University Hospital, Translational Research Network of Pediatric Infectious Diseases (RITIP), 28049 Madrid, Spain; tsainzcosta@gmail.com (T.S.); lucia.esc.tar@gmail.com (L.E.); soniaalcolearuiz@gmail.com (S.A.); lidia.madrid.99@gmail.com (L.P.); ccalvorey@gmail.com (C.C.); 2 Microbiology Department, La Paz University Hospital, 28049 Madrid, Spain; falces88@gmail.com; 3 Pediatrics, Infectious Diseases, Rheumatology and Immunology Department, University Hospital Virgen del Rocío, 41013 Sevilla, Spain; beadefelipe@gmail.com (B.d.F.); olafneth@gmail.com (O.N.)

**Keywords:** SARS-CoV-2, serology, antibody, COVID-19, children, pediatric

## Abstract

Background: Antibody dynamics over time after SARS-CoV-2 infection are still unclear, and data regarding children are scarce. Methods: A prospective cohort study was performed including children infected by SARS-CoV-2 between March and May 2020. Patients were categorized into 3 groups: children admitted with COVID-19; outpatient children with mild COVID-19; and seropositive children participating in a seroprevalence study among cohabitants of infected healthcare workers (HCWs). Six months after the infection, a new serological control was performed. Results: A total of 58 children were included, 50% male (median age 8.3 [IQR 2.8–13.5] years). The median time between the two serological studies was 186 (IQR 176–192) days, and 86% (48/56) of the children maintained positive IgG six months after the infection. This percentage was 100% in admitted patients and 78% among the rest of the included children (*p* = 0.022). The diagnoses of lower respiratory tract infection and multisystemic inflammatory syndrome were associated with persistence of IgG (*p* = 0.035). The children of HCWs in the seroprevalence study lost antibodies more often (*p* = 0.017). Initial IgG titers of the children who remained positive six months after the infection were significantly higher (*p* = 0.008). Conclusions: Most children infected by SARS-CoV-2 maintain a positive serological response six months after the infection. Those children who lost their IgG titer were more frequently asymptomatic or mildly symptomatic, presenting with low antibody titers after the infection.

## 1. Introduction

One year after the start of the SARS-CoV-2 pandemic, understanding the dynamics of the humoral immune response to the virus remains a challenge. A myriad of studies have attempted to characterize proper COVID-19 immune response both among children and adults.

Despite the relatively short period of time since the identification of the disease, there is evidence to support that certain individuals maintain persistent antibodies after infection, whereas in most patients, antibody titers show a progressive decline over time [1,2,3]. In addition, some authors have underlined that eight months after infection, anti-nucleocapsid antibodies remain more frequently positive in severe patients compared with those with mild symptoms [2]. Most authors highlight that disease severity underlines the magnitude of the immune response, with significantly higher antibody titers among adults with severe COVID-19 [3,4,5,6].

The role of antibody persistence in the development of long-lasting protection against the virus is controversial. However, to understand long-lasting immunity after the infection, a proper characterization of humoral immune response is required both among children and adults.

SARS-CoV-2 infection is frequently paucisymptomatic or even asymptomatic among children. Not surprisingly, serology-based studies including children have described seroprevalence rates higher than expected among children at risk, such as among children of healthcare workers (HCWs) [7]. However, longitudinal follow-up studies evaluating the dynamics of humoral response in the pediatric population are scarce, although some authors have observed important differences when comparing children and adults in terms of antibody titers [8]. In this study, we aimed to characterize the humoral response over time among children presenting with different severities of symptoms after being infected by SARS-CoV-2. Thus, we assessed the persistence of antibodies against SARS-CoV-2 six months after the infection and identified factors associated with antibody persistence.

## 2. Materials and Methods

We performed a prospective cohort study at La Paz University Hospital between May and December 2020. We invited children with a positive SARS-CoV-2 antibody test between May and June 2020 to participate in the longitudinal cohort. We included participants who had a positive SARS-CoV-2 antibody test between May and June 2020 and categorized them into 3 groups of patients according to clinical presentation: children admitted with COVID-19; children under follow-up at the outpatient clinic with mild forms of disease; and children participating in a seroprevalence study among cohabitants of HCWs infected by SARS-CoV-2, who had experienced a mild or asymptomatic condition [7].

We collected epidemiological and clinical data by reviewing medical records and through a brief survey specifically designed for the study, including presence of clinical symptoms, hospitalization, duration of the stay, and admission to the intensive care unit.

All participants underwent longitudinal follow-up, and we performed a second serological study (immunoglobulin G, IgG) 6 months after baseline. We performed a SARS-CoV-2 IgG test employing an enzyme-linked immunosorbent assay (ELISA) (COVID-19 ELISA IgG, Vircell, Granada, Spain). Among the children of HCWs, the original protocol included baseline determination of IgA; thus, a second determination was performed using the ELISA test for IgA detection (Anti-SARS-CoV-2 IgA, Euroimmun, Lubeck, Germany). Results were interpreted according to the manufacturer’s instructions.

The study protocol was evaluated and approved by the hospital’s ethics committee (PI-4324), and an informed consent/assent document was signed by the parents/legal guardians and by all the patients older than 12 years of age.

The qualitative data were expressed as absolute and relative frequencies and the quantitative data as median and interquartile range (IQR). We compared the categorical variables employing the chi-squared and Fisher’s exact tests and the continuous variables with Student’s t-test or nonparametric tests as appropriate. We considered a 2-tailed value of *p* < 0.05 as statistically significant and performed all analyses with the Statistical Package for the Social Sciences, version 21.0 (IBM Corp., Armonk, NY, USA).

## 3. Results

A total of 58 children with a median age of 8.3 (IQR 2.8–13.5) years were included in the study and completed the follow-up; 50% were male. The median time between the two serological studies was 186 (IQR 176–192) days. Of 58 participants, 21 (36.2%) were patients under follow-up after admission due to COVID-19; 9 (15.5%) were followed at the outpatient clinic because of mild COVID-19; and the remaining 28 (48.2%) were children of infected HCWs who turned out to be seropositive after the first pandemic wave. According to the medical history, 86.2% of participants presented symptoms and 36.2% required hospital admission.

Lower respiratory tract infection (LRTI), including pneumonia, was the most common diagnosis among the patients with symptoms (11/50; 19%), followed by fever without a source (11/50; 19%), upper respiratory tract infection (9/50; 15.5%), and multisystemic inflammatory syndrome in children (MIS-C) (7/50; 12.1%). The clinical characteristics of the hospitalized children and the outpatients are described in Table 1.

Six months after a positive serology, 86.2% (50/58) of the children remained positive for SARS-CoV-2 IgG antibodies. This percentage increased to 100% among the children who required admission and decreased to 78.4% if only non-admitted children were included in the analysis (odds ratio [OR] 1.7; 95% CI 1.3–2.1; *p* = 0.022). Among the other diagnoses, LRTI and MIS-C were associated with persistence of IgG at six months (OR 1.6; 95% CI 1.2–1.9; *p* = 0.035). The children of HCWs who participated in the seroprevalence study (group 3) lost antibodies more often (OR 2.08; 95% CI 1.3–2.1; *p* = 0.017). The initial IgG titers of the children who remained positive six months after the infection were significantly higher than those initially observed in the children who finally lost them during the follow-up screening (48.5 ± 25.2 vs. 18.9 ± 20.3; *p* = 0.008).

No differences were found regarding the persistence of IgG according to sex, and no association between antibody persistence and age could be identified. No reinfections were registered during follow-up.

Serial IgA was determined in 27 children (all HCWs’ children) and was initially positive in 14 (51.9%). At six months, it was positive in 18/26 (69.2%). Only one patient lost IgA titers.

## 4. Discussion

Dynamics of antibodies over time after SARS-CoV-2 infection are still unclear, and data regarding children are scarce. We observed that most children infected by SARS-CoV-2 maintained a positive serological response six months after the infection. All the children who required hospital admission remained seropositive, whereas one out of five children presenting with mild or asymptomatic infection, especially those presenting with low antibody titers, lost their antibodies. IgA titers were initially present in half of the children studied (all mild or asymptomatic) and persisted as positive six months later.

Our results are encouraging, given that most participants maintained antibody titers six months after infection. Although the clinical significance of these findings is uncertain, the generation of neutralizing antibodies is typically correlated with protective immunity [9]. Our results are in line with previous findings in adult populations, in which SARS-CoV-2 IgG persisted six months after the infection in most participants [1,2,3,5,10,11]. Data are scarce regarding the long-term humoral response in children [8,12]; however, some pediatric series report a rate of antibody persistence below 30% after three months of infection. The use of a test measuring the production of anti-nucleocapsid (N) antibodies [12] could account for the differences found. The immune response of children is mainly based on antibodies against the spike (S) protein, which the virus uses to enter cells, while antibodies against the N protein, essential for virus replication, are lacking. This lack of data could reflect the fact that children do not generally experience widespread infections [13]. In our study, we have assessed the production of anti-S plus anti-N antibodies. As described among adults [3,5,6], an antibody decrease was mostly observed among the asymptomatic or mildly symptomatic patients in our study. In addition, some authors have reported that children present lower titers of anti-SARS-CoV-2-specific antibodies than adults [8,9], including S protein, presenting reduced neutralizing activity compared to adults [9]. These findings are interesting, given that children are typically less affected by SARS-CoV-2 than adults, presenting mild or asymptomatic infections, with lower rates of seroconversion, especially among infants and young children [7,14,15,16].

Several authors have suggested that this low seroprevalence might not reflect the real rate of infection [2,17,18] among children, but the immune response of children probably differs from that in adults. Evaluating seroconversion using assays that measure anti-N antibodies could underestimate the real incidence of the infection in children. A recent study by Hachim et al. analyzing long-term humoral responses in children reported lower anti-S and anti-N antibodies in children compared with adults, but higher presence of antibodies against other nonstructural viral proteins (open reading frames, ORFs), which act as interferon antagonists, although these are not routinely analyzed by conventional serological tests [8]. Children with mild or asymptomatic infections could present with high antibodies against interferon antagonists but lower antibodies toward structural proteins, tending to lose them over the time, whereas children with moderate-severe COVID-19 might present a humoral response similar to infected adults, with high and persistent S antibody titers, as we have observed. Our study supports previous ones suggesting that seroprevalence studies in children are needed to better estimate the real prevalence of COVID in children, as many may have been missed for clinical mild symptoms or false-negative PCR tests [19,20]. Although PCR has been considered the gold standard to confirm the infection, false-negative results could be observed in children with low viral load in respiratory samples, asymptomatic individuals, or low-quality sampling, as we have observed in our cohort.

Critically ill adults rapidly seroconvert, presenting with high IgG and IgA titers, although paradoxically, this humoral response does not necessarily correlate with a better outcome. Some recent data support that asymptomatic children presenting with mild disease can mount an innate and local mucosal immune response able to neutralize SARS-CoV-2 without seroconversion [13,21,22], which could explain their mild clinical presentation [9]. Some authors have suggested that IgA titers strongly correlated with neutralizing antibodies [23]. In our series, IgA titers, present in half of the children studied, all asymptomatic or with mild disease, persisted in most after six months. Garrido et al. have recently reported that up to 79% of infected children in their series presented positive IgA titres four months after the infection [24]. We hypothesize that this finding could reflect a strong mucosal immune response in these participants. Other authors have reported IgA positivity in children and young adults who developed SARS-CoV-2-related chilblain-like lesions without IgG seroconversion [25,26], suggesting a mucosal immune control of the infection that might impair the triggering of an IgG response. In contrast, large studies performed in adults reported that only 37% of patients remained IgA-positive six months after the infection [5].

Unfortunately, methodologies differed widely among the studies, which used different antibodies (IgG, IgM, and/or IgA) targeting different viral proteins, such as S or N antigens, whose correlation with viral neutralization is unclear [27]. Different sensitivities and technologies, such as lateral flow immunochromatography, ELISA, or immunofluorescent antibody [27,28], complicate the interpretation of results. However, we believe all these data suggest different immune responses against SARS-CoV-2 between adults and children and between mild and severe infections.

Our study has several limitations, including the relatively small sample size. However, we analyzed the long-term humoral response across the entire clinical spectrum of the disease in children, studying asymptomatic children, patients with mild disease, and those with moderate/severe infections requiring hospitalization. IgA detection was only performed in a group of children with asymptomatic/mild infection. We did not study other antibodies, such as anti-spike receptor-binding domain or anti-ORFs. Finally, we did not perform neutralization assays, which likely provide more relevant data regarding protective humoral immunity. However, many authors have observed that antibody titres strongly correlated with neutralizing activity and immune protection [23,24,29,30]. Patil et al. observed that IgA and IgG antibodies correlated stronger with neutralizing antibody [23]. Dogan and colleagues also reported a positive correlation between SARS-CoV-2-specific antibody levels and neutralization activity in a study of 115 adult participants [29]. Wu et al. reported that long-term antibody responses correlate closely with the capacity to neutralize SARS-CoV-2 in infected patients [30]. In paediatric population, Garrido et al. observed similar levels of SARS-CoV-2-specific IgM, IgG, and IgA antibodies and neutralizing activity up to four months after acute infection in asymptomatic and mildly symptomatic children, with higher neutralizing activity compared to adults [24]. Although we did not perform neutralization assays, as correlation between neutralization activity and antibody levels have been extensively reported, our findings strongly suggest that children may generate a more robust and durable humoral immune responses than those of adults, as other authors have observed [24].

Despite its limitations, long-lasting humoral response in pediatric COVID-19 has scarcely been reported, especially among moderate or severely ill children. In our study, we observed that most children maintained a positive serological response six months after the infection, and those who lost their IgG titer were less symptomatic, presenting with low antibody titers after the infection. However, there is a need for longer follow-up of children infected by SARS-CoV-2 in order to establish the length of humoral protection in this population. Further studies are needed to elucidate the role of long-term humoral responses in children after SARS-CoV-2 infection and its association with protection against reinfections.

## Figures and Tables

**Table 1:** Characteristics of hospitalized and outpatient children infected by SARS-CoV-2.

	Admitted Children (*n* = 21)	Outpatients (*n* = 37)	*p*
Sex (male)	14 (66.6%)	15 (40.5%)	0.056
<1 year old	8 (38%)	3 (8%)	0.005
Age (years)	6.5 (IQR 0.8–12)	10.1 (IQR 5.2–13.6)	0.133
PCR positive	13/21 (61.9%)	4/12 (33.3%)	0.000
Health care workers’ children	0	28	0.000
Diagnosis			0.000
- Asymptomatic	0	8 (21.6%)	
- URTI	3 (14.2%)	6 (16.2%)	
- LRTI	9 (42.8%)	2 (5.4%)	
- FWS	10 (47.6%)	1 (2.7%)	
- MIS-C	7 (33.3%)	0	
- Other (GI mainly)	1 (4.7%)	11 (29.7)	
6th month IgG positive	21 (100%)	29 (78.3%)	0.022

PCR, polymerase chain reaction; URTI, upper respiratory tract infection; LRTI, lower respiratory tract infection; FWS, fever without a source; MIS-C, multisystemic inflammatory syndrome in children; GI, gastrointestinal.
